# Association between Periodontal Disease and Alzheimer's Disease Risk Factors: A Longitudinal Oral Microbiome Study

**DOI:** 10.1002/alz70856_103436

**Published:** 2025-12-26

**Authors:** Irene Yang, Kevin Hendler, Frank A. Scannapieco, Gabrielle Boykins, Whitney Wharton

**Affiliations:** ^1^ Emory University, Atlanta, GA, USA; ^2^ The State University of New York, University at Buffalo, Buffalo, NY, USA

## Abstract

**Background:**

Evidence suggest an association between periodontal disease (PerioD) and Alzheimer's disease (AD), with PerioD‐associated microbial ecosystems driving oral and systemic inflammation that may activate or accelerate neuroinflammation, a hallmark of AD. Social determinants of health (SDoH) are critical factors influencing both oral health and AD risk yet are often overlooked, and rarely investigated together. This study aims to characterize and compare the oral microbiome of age‐ and education‐matched individuals at high risk for AD by virtue of family history, with and without PerioD, and to investigate the relationships between PerioD‐associated microbiome features, SDoH, systemic inflammation and brain inflammation, and AD biomarkers (in cerebrospinal fluid [CSF]).

**Method:**

This two‐year NINDS‐funded study collects oral microbiome samples, blood, and CSF annually in a cognitively normal, racially diverse cohort (*n* = 165). Metagenomic sequencing will be used to investigate cross‐kingdom microbial communities and their association with inflammatory and systemic markers. Surveys and interviews investigate behaviors and SDoH influencing PerioD and AD risk.

**Result:**

To date, 55 participants have been recruited. Participants are 62 years of age on average, predominantly white (70%), female (63.3%), with Stage 1–2 periodontitis (85.7%). Preliminary analyses found no significant relationships between bleeding on probing, behavioral factors, SDoH variables, and Montreal Cognitive Assessment (MoCA) scores, which was expected given the small sample size. As recruitment continues, we anticipate identifying associations between oral microbiome features, inflammatory markers, AD biomarkers and cognitive outcomes. SDoH, such as access to dental care and oral hygiene behaviors, may mediate these relationships, offering insights into the interplay between periodontal disease, systemic inflammation, and AD risk.

**Conclusion:**

By leveraging longitudinal data and exploring upstream sociocultural factors, this research addresses critical gaps in understanding PerioD's contribution to AD risk. Findings will provide novel insights into the interplay between the oral microbiome, systemic inflammation, brain inflammation, and AD risk.

